# The experiences of family caregivers living with breast cancer patients in low-and middle-income countries: a systematic review

**DOI:** 10.1186/s13643-020-01408-4

**Published:** 2020-07-23

**Authors:** Grace Kusi, Adwoa Bemah Boamah Mensah, Kofi Boamah Mensah, Veronica Millicent Dzomeku, Felix Apiribu, Precious Adade Duodu, Bakinam Adamu, Pascal Agbadi, Kwadwo Osei Bonsu

**Affiliations:** 1 Department of Nursing, Faculty of Allied Health Sciences, College of Health Sciences, Kwame Nkrumah University of Science and Technology, Kumasi, Ghana; 2 Department of Obstetrics and Gynecology, Komfo Anokye Teaching Hospital, Kumasi, Ghana; 3 Oncology Directorate, Komfo Anokye Teaching Hospital, Kumasi, Ghana; 4 Department of Pharmacy Practice, Faculty of Pharmacy and Pharmaceutical Sciences, Kwame Nkrumah University of Science and Technology, Kumasi, Ghana

**Keywords:** Breast cancer, Family caregiver, Low- and middle-income countries, Experiences

## Abstract

**Introduction:**

Caregivers of women with breast cancer in low-and-middle-income countries experience significant physical and economic burdens. The review aimed to map the evidence of studies that had reported on the experiences of family caregivers of women diagnosed with breast cancer.

**Methods:**

A systematic literature search was conducted in CINAHL, PubMed, PsycINFO, Scopus, and Web of Science databases using a combination of key search terms and medical subject heading terms such as “family caregiver,” “breast cancer,” “home care,” “low-and-middle-income countries,” “experience,” “effect,” and “coping mechanism.” A total of 1781 articles were retrieved and screened. Nineteen studies addressing caregiving experiences were included in the final review based on the inclusion and exclusion criteria.

**Results:**

The systematic review yielded 19 studies that focused on caregivers’ motivation, needs of caregivers, intervention for caregivers, and consequences of caregiving. The most significant correlates of the quality of life among caregivers were disease severity, functional status of patients, and family income. The challenges encountered by caregivers were mostly psychosocial.

**Conclusions:**

Caregivers play a crucial role in the management of women with breast cancer. However, they are faced with increasing challenges in their caregiving roles. Understanding the nature and extent of the burden experienced by family caregivers in developing countries will facilitate the development of appropriate interventions that can help improve caregivers’ quality of life. Gaps in recent studies were identified, and suggestions for future research were also addressed in this review.

**Systematic review registration:**

PROSPERO CRD42019118391

## Introduction

The increasing incidence and mortality rate of breast cancer has produced challenges in caring for women with breast cancer, especially in low- and middle-income countries (LMICs) [1, 2]. This challenge has implications for relatives and friends who become family caregivers [3]. Breast cancer treatment is now shifting from an inpatient setting to a more outpatient setting due to contextual barriers to oncology services that exist in LMICs, such as limited availability of treatment facilities, lack of cancer specialists [4], and limited geographical access to oncology care [5]. This shift has resulted in the role of family caregivers as significant members of the cancer care system who are expected to provide physical, emotional, financial, and psychosocial support to women diagnosed with breast cancer in the home setting [6–9]. However, according to Khanjari et al. [10], the current declining socioeconomic trends in LMICs potentially alter the capacity of caregivers to meet the growing demand for home care support for women with breast cancer.

The burden of breast cancer caregiving includes emotional distress, financial burden, physical stress, and fear of uncertainty among caregivers [11–13]. Furthermore, the advanced stage disease presentation, a hallmark of this disease in LMICs, can result in increased psychosocial morbidity, poor physical health, and overall poor quality of life among caregivers [6, 10, 11, 14]. Hashemi-Ghasemabadi et al. [6] have indicated that caregivers who deliver care to women with breast cancer in LMICs experience unique challenges due to under-resourced and limited cancer support systems. For instance, few West African studies focusing on this phenomenon have highlighted that challenges encountered by caregivers include loss of job, difficulty in balancing multiple roles, a decline in physical health, lack of access to healthcare funding, emotional trauma, and lack of information in managing breast cancer-related symptoms such as wound and lymphedema [4, 15]. A recent Ghanaian study reporting on the motivation and caregiving experiences of family caregivers of advanced breast cancer patients showed that sociocultural obligation and reciprocity were the main reasons for assuming the caregiving role [4]. Further, the study also highlighted that caregivers provided multi-dimensional forms of support such as physical, psychosocial, emotional, financial, symptom management, and spiritual support for women living with advanced breast cancer. Financial burden through the provision of out-of-pocket money for treatment costs and other related non-medical costs were the main challenge reported by participants in this study [4]. The study recommended home-based support programs and direct governmental social intervention programs to assist caregivers in their caring role.

However, to date, no systematic review of family caregiving in breast cancer that is specific to LMICs has been conducted to fully understand the experiences and challenges faced by this group of caregivers. Exploring the experiences of family caregivers is critical in providing potential interventions that can aid in addressing the needs of family caregivers in LMICs. Hence, the rationale of this systematic review was to summarize and appraise existing evidence on studies that had reported on:

- Home care experiences of caregivers of women diagnosed with breast cancer in LMICs.
- Effects of caregiving on the family caregivers in LMICs.
- Coping mechanisms utilized by family caregivers of women living with breast cancer in LMICs.

## Materials and methods

The protocol of this review is duly registered (CRD42019118391) in the PROSPERO international prospective register of systematic reviews. Studies that explored the experiences of caregivers living with breast cancer patients in LMICs were searched.

### Inclusion and exclusion criteria

All studies (quantitative studies, qualitative studies, and mixed-method studies) were conducted in diverse settings such as hospitals or communities published from January 2000 to March 2020. Other criteria for inclusion were (1) family caregivers of breast cancer patients aged 18 years and above, (2) providing non-paid caregiving services to breast cancer patients, (3) full-text published articles from LMICs (low-and-middle-income country was operationalized in this study as low-, lower-middle, and upper-middle-income economy based on the January 2020 World Bank list of analytical income classification of economies) [16], (4) reporting on family caregivers experiences, and (5) articles published in the English language.

### Exclusion criteria

Studies were excluded from this review based on the following criteria: (1) focused on paid and formal caregivers such as healthcare professionals; (2) not published in the English language (due to limited availability of translation service to the authors); and lastly, (3) systematic reviews, abstracts, editorial reports, letters, conference articles, and gray literatures with no full-text published articles were excluded because they were not considered as scientific published articles.

### Search strategy

A systematic review was conducted according to the Preferred Reporting Items for Systematic Review and Meta-Analysis Protocols (PRISMA-P). We conducted a comprehensive search of qualitative, quantitative, and mixed-methods literature that was published from January 2000 to March 2020 in the electronic databases, i.e., PubMed, CINAHL, Scopus, Web of Science, and PsycINFO, to retrieve all English language literature that contained information on family caregivers of breast cancer patients in low- and middle-income countries. As previously defined, studies were defined into “low-income,” “lower-middle-income,” and “upper-middle-income” countries as categorized by the World Bank [16]. Primary concepts such as “family caregiver,” “breast cancer,” “home care,” “low-and-middle-income-countries,” “experience,” “effect,” and “coping” and their Medical Subject Headings (MESH) were used for the search (Additional file 1: Table S1). The subject search and text word search were performed separately in all the databases and then combined with Boolean operators “OR” and “AND.” Combined terms used, for example, were (“Caregiv*(MESH)” OR “Family caregiv (MESH)*”) AND (“Breast cancer (MESH)” OR “Breast neoplasm*(MESH)”) AND (“low-income countr*[tw]” OR “lower-middle-income countr*[tw]”). Additional articles were searched from other sources such as Google Scholar as well as hand-searching the references of all included studies. This produced three additional articles from Google Scholar [17–19].

### Literature screening

Following the literature search, citations were imported into Mendeley Desktop (version 1.19.4) reference manager for storage and screening of articles as well as the removal of duplicates. The remaining articles were then screened independently by titles and abstracts. Two reviewers (GK and KBM) screened all the citations that were identified from the search using standard systematic review procedures (inclusion and exclusion criteria). Any disagreement was resolved by discussion and consensus or by consulting a third reviewer (ABBM) where needed.

### Quality appraisal or assessment tool

The quality of the selected studies was assessed independently by GK and KBM using the Mixed Methods Appraisal Tool (MMAT) version 2018 [20]. This tool has been developed to evaluate qualitative, quantitative, and mixed-method studies using two screening questions and four methodological criteria. The tool assesses the appropriateness of the aim of the study, adequacy and methodology, study design, participant recruitment, data collection, data analysis, presentation of findings, authors’ discussions, and conclusions [21]. Each question carries three possible responses: “yes,” “no,” or” can’t tell.” A star is assigned (four stars maximum possible score) to each yes response and is converted to percentages (from one star = 25% to four stars = 100%) [20]. Using the aforementioned domains, score from 0–25% is regarded as weak, 50% is regarded as moderate, 75% is regarded as moderate-strong, and 100% is regarded as strong [20].

The interrater reliability for each MMAT dimension for quality ratings of the included studies was evaluated using Cohen’s kappa statistic [22]. This was done to assess the level of agreement of quality ratings between the two independent authors (GK and KBM) who performed the quality assessment for the included studies. The interrater reliability of each MMAT dimension was assessed and reported as Cohen’s kappa coefficient and 95% confidence interval (95% CI) with two-sided *p* value. Acceptable kappa values 0.80–0.90, > 0.90, and 1.0 represent strong, almost perfect, and excellent levels of agreement between raters [22].

### Data extraction and analysis

Two reviewers (GK and KBM) independently extracted key characteristics from the included articles: bibliographic information (authors’ name, year of publication, country, and address). Others included the methodology (study aim, design, setting, and sample size), caregivers’ characteristics, and major findings on caregivers’ experiences, conclusions, and limitations of the study as described in Table 1. There was significant heterogeneity in the measurement of the outcomes of the eligible studies, making it impossible to pool data in a meta-analysis. Therefore, a narrative analysis or strategy was employed to synthesize the evidence.

**Table 1:** Summary of study characteristics and findings

Author/year	Country	Aim/research questions	Study design	Caregivers	Findings	Conclusion	Limitation
Vahidi et al. (2016) [3]	Iran	Determine factors associated with caregiver burden among primary caregivers of women with breast cancer	Descriptive correlation study	150 primary caregivers; caregivers mean age 39.60 years; 77 (51.3%) were males	Caregivers assisted with activities of daily living, administering medication, symptom management, and financial support. Higher caregiver burden was associated with increasing assistance with activities of daily living, low educational level, gender, and poor financial status	Caregivers must be provided with comprehensive care needs support. Primary caregivers need to be supported financially by relevant organizations, such as government agencies and charities. Other factors such as dedicating a place for caregivers in the clinic to receive care skills training from expert nurses can be effective	Cross-sectional studies do not show the difference in burden in response to cancer progression in the patient; therefore, longitudinal studies are recommended
Gabriel, Aluko and Okeme (2019) [15]	Nigeria	Impact of caregiving burden on the informal caregivers of women with breast cancer	Descriptive study	118 caregivers; mean age = 41.9 years. Most were child (77.1%), spouse (14.4%), and others (8.5%)	Determinants of high caregiver burden were payment type for treatment, family income, relationship to the patient, social support, and self-efficacy	Strategies to improve self-efficacy such as additional training, follow-up, peer education, and support groups for caregivers might decrease the caregiver burden	Use of convenience sampling making it difficult to identify causal relationships
Zhu et al. (2014) [31]	China	To assess the quality of life in male spouse caregivers	Cross-sectional study	243 spouse caregivers. Mean age = 49.5 years	Decreasing patient functional status was significantly associated with poor quality of life among caregivers. High family income and longer sleeping time increased the quality of life among caregivers	Attention should be paid to male spouse caregivers as a separate group. Nurses can understand the status of caregiver burden and QOL by assessing both patients and spouses. The mental health of spouse caregivers was disrupted more seriously than the physical health	The study focused on spouse caregivers and hence cannot be generalized for other non-spousal caregivers
Hashemi-Ghasemabadi et al. (2016) [6]	Iran	Transition to the new role of caregiving for families of women with breast cancer diagnosis	Qualitative descriptive exploratory study	23 caregivers. Mean age = 37.5 years. 69.6% were females and 30.4% men	Emerged themes: •“Grasping a new situation without preparation” •“Perceived inefficiency” •“Infinite absence” •“Abandoned in the role” Caregivers cited that they were unprepared for their new role and did not have the necessary skills and knowledge to manage breast cancer and its related symptoms. Caregiving was also described as a time-consuming role which competed with other family roles and responsibilities.	By understanding their experiences in the transition to the new role, it is possible to provide detailed information for designing evidence-based healthcare interventions and comprehensive family-centered care program. Also, interventions can be tailored to the objectives and resource limitations, for the support and resolution of the challenges of caregivers to decrease the negative consequences of caregiving	Study could not be generalized because it is a qualitative study. Provided limited information on coping strategies
Sahadevan et al. (2019) [26]	India	Identify the determinants of depression among caregivers of patients with breast cancer	Cross-sectional survey	384 caregivers. Mean age = 47.25 years. 163 (42.4%) were males	Higher financial responsibilities, being a spousal caregiver are important determinants of depression among caregivers	Cancer specialists in treatment team need to be aware of the need for psychological assessment of principal caregivers. They should be trained to pick up depressive symptoms and its associated risk factors at the earliest and should be offered services to the needful. This approach ultimately improves the outcome of cancer treatment	This cross-sectional survey was a hospital-based study and may not be generalizable to all caregivers in India
Jaafar et al. (2014) [24]	Malaysia	To examine the rates of clinical depressive disorders in caregivers during breast cancer treatment	Cross-sectional study	130 caregivers comprising of spouses (46.9%), children (40.0%), and siblings (13.1%)	Depressive disorders were related to low educational status of caregivers and high duration of caregiving	This finding gives clues to intervening depression among the caregivers by providing support in the form of respite care to the caregivers and improving engagement of the caregivers by the health care providers. The results should increase the health care providers’ awareness of the vulnerability of this population and discard a patient-centric approach of treatment	Data on coping mechanism were missing in this study. The study was conducted in a single facility which might limit generalizability to similar population
Gabriel and Mayers (2019) [27]	Nigeria	To assess the effectiveness of a psychosocial intervention in reducing caregiver burden among caregivers	Quasi-experimental study	108 caregivers; intervention group (54); control group (54). Caregivers were primarily spouse (30.6%), parent (15.7%), sibling (17.6%), child (21.3%), and friend (14.8%)	Primary caregivers who received the psychosocial intervention reported significant decrease in burden at 6 weeks and 12 weeks. Further, the psychosocial intervention also improved the overall quality of life of caregivers	The need for effective advocacy on the issue of caregiver burden is vital. Relevant stakeholders in the healthcare sector, especially in palliative care, should conduct advocacy campaigns to promote the culture of caring and support for the person with cancer and the caregiver	Non-randomized deign was used to recruit participants. Psychosocial intervention did not address the subjective aspect of the caregiver burden
Giray and Akyuz (2019) [28]	Turkey	To assess relationships between caregiver burden, quality of life, arm disability, grip strength, and lymphedema symptoms in patients with postmastectomy lymphedema	Prospective cross-sectional study	52 caregivers. Mean age = 48.46 years. 14 (26.9%) were females and 38 (73.1%) were males	Caregiver burden was associated with arm disability and quality of life of these patients. Arm disability affects caregiver burden and quality of life in these patients. Arm disability was higher in patients at stage 3 lymphedema than patients at milder stages	Arm disability should be diagnosed and treated to improve caregiver burden and quality of life	This was an observational study using a convenience sample. Comparison of caregiver burden before and after lymphedema development and treatment can more enlighten the importance of caregiver burden in the management of patients with postmastectomy lymphedema
Moreno-Gonzalez et al. (2019) [29]	Mexico	To describe the experience of family care of women with breast cancer during treatment from the perspective of caregivers	Qualitative study	Seven caregivers (3 men and 4 women)	Male caregivers stated that the absence of a breast did not interfere with the perception of their femininity or sexual attractiveness. Caregivers experienced fear and despair for not knowing how to alleviate women symptoms. Also, caregivers experienced fear of the unknown and sometimes anger. They also looked for strategies to maintain their emotional balance. Female caregivers living this experience expressed a greater perception of the risk of suffering from breast cancer, which favored their self-care by knowing about the timely detection	The experience of caregivers of women with breast cancer generated profound changes in them through the discovery of their reach and limitations in difficult situations	The study leaves aside different contexts and events such as rupture between couple and family disintegration that may prevail
Wulandari et al. (2017)	Indonesia	Determine the experience of stress and adaptation of breast cancer patient’s family	Qualitative method	7 male caregivers	Caregivers cited that they experience stressors related to difficulty in managing of the disease and the financial needs of the patient. Caregivers further reported that financial problem became a stressor from the beginning until the end of the breast cancer treatment. Components of caregiver coping mechanism consisted of strategies such as emotional support from health professionals, prayer, and gratitude	The experience of stress and adaptation of the family of breast cancer patients is a continuous stage. Continued coping such as support from professional nurses may be needed to minimize the stress and improve adaptation of family caregivers	Caregivers in this study was small. Study did not explain comprehensively measures that were undertaken to ensure trustworthiness of the study
Bahrami and Farzi (2014) [18]	Iran	Determine the effect of a supportive educational program on the caregiving burden and quality of life in the family caregivers of women with breast cancer	Two‑group two‑step before–after clinical trial	64 family caregivers. Mean age (control group) = 38.97 Mean age (experimental group)	The study implemented a supportive educational program to a group of caregivers. After the intervention has been implemented, the results showed that in the experimental group, the mean score of physical, mental, spiritual, and environmental domains and overall quality of life in the family caregivers was significantly increased compared to the control group. Further, in the experimental group, the mean score of caring burden among the caregivers was significantly decreased compared to the control group	The findings of the study suggested that supportive educational program can improve physical, psychological, spiritual, and environmental domains and overall quality of life. It can also decrease the caring burden in the family caregivers of women with breast cancer	Small sample size was used
Mahadevan et al. (2013) [32]	Malaysia	To determine the proportion of stress among the caregivers of breast cancer patients receiving oncologic treatment at Kuala Lumpur Hospital and to determine the predictors of stress among the caregivers	A cross-sectional study	130 caregivers with mean age of 42.8 ± 14.5 years	Generally, caregivers had higher levels of stress. Approximately 16% of caregivers felt emotionally strained and 26% acknowledge that taking care of the patient is hard on them emotionally. In addition, caregivers felt less in control of their lives. Caregivers who looked after older patients were less likely to be stressed	There should be awareness among medical personnel about the high likelihood of stress among the family caregivers of breast cancer patients and a heightened sensitivity to the caregivers’ emotional condition. Caregivers should have easy access to mental health services. There should be respite care facilities in order to relieve the caregivers from constant caregiving burden and stress	The study was cross-sectional in design, whereby being conducted at one point of time; no causal relationship can be inferred between the outcomes and the variables. The application of non-random sampling method within a convenience samples frame could create sampling bias, resulting in over- or under-representation of certain members of the study population
Yeung et al. (2018) [30]	China	This study aimed to examine the experience of guilt and its correlates among Chinese husbands of women with breast cancer	A cross-sectional survey	176 husbands caregivers with mean age of 50.22 years	Lower endorsement of the “masculinity strength” gender-role norm, and higher caregiving burden and social support seeking were associated with higher caregiving guilt. Unexpectedly, higher marital satisfaction and less protective buffering were associated with higher caregiving guilt. Younger husband caregivers in our sample were more likely to report higher guilt. Also, caregiving burden was associated with caregiver guilt. Also, protective buffering and caregiver guilt was conditional to caregiver’s level of marital satisfaction	The new findings and complex interplay between caregivers’ characteristics (including endorsement of male gender-role norms and marital satisfaction) and coping strategies (protective buffering and seeking social support) in predicting guilt imply that individual and cultural characteristics may change the effectiveness of specific coping strategies in cancer caregivers’ well-being	Caregivers were recruited from two hospitals. Hence, sample may not be representative of the total population of caregivers of breast cancer patients
Heidari Gorji et al. (2012)	Iran	To examine the correlates of depression in relation to quality of life among breast cancer caregivers	A cross-sectional descriptive design		Findings were demonstrated that high percent of caregivers were afflicted by mild and moderate depression. The results showed that 42 and 11% reported moderate and low quality of life, respectively. The study demonstrate that psychological issues have a significant impact on quality of life	The study demonstrate that psychological issues have a significant impact on quality of life. Additionally, help and attention to caregivers would be beneficial in improving quality of life of all family of patients	This is a cross-sectional study; hence, results may not be generalizable to all caregivers in Iran
Yuanyuan An et al. (2019)	China	To identify the influence of family caregiver’s burden on breast cancer patient’s QoL and possible mediators	A cross-sectional design	382 caregivers	Higher level of family caregiver’s burden was associated with higher levels of patient’s anxiety and depression	Given the important role of family caregiver’s burden, it should be targeted by the intervention aiming to improve breast cancer patients’ QoL and well-being	The data were cross-sectional, which precludes conclusions regarding causation and the direction of relationship among variables. Second, the findings are only generalizable to the population studied
Nejad et al. (2016)	Iran	Determine and compare the caregiver strain index scores of breast cancer informal caregivers, before and after a patient-caregiver educational and telephone follow-up program	Experimental study design	60 caregivers Mean age > 30 (28.3 years) 30–50 (43.3 years) < 50 (28.3 years)	The mean caregiver strain score of the intervention group was 8.3 ± 2, and it dropped to 4.8 ± 2.3 post-intervention	Caregiver burden decreased significantly in the intervention group after the patient-caregiver education and follow-up program (*P* < 0.001)	The intervention period was relatively short, thus limiting the generalizability of the results
Din et al. (2017) [25]	Malaysia	To determine the anxiety disorders specifically focusing on the family caregivers of breast cancer patients	A cross-sectional study	130 caregiver-patient dyads	The study found that more than a 10th (11.5%, *n* = 15) of the family members who were primarily involved in caring for breast cancer patients had anxiety disorders. Specifically, 8 caregivers (6.1%) had generalized anxiety disorder, 6 were (4.6%) diagnosed to have panic disorder, and one (0.8%) had social phobia associated with both the patients’ type of treatment and non-shared caregiving	A multidisciplinary management approach should be extended to those at risk which would directly and inadvertently optimize the treatment care for patients with breast cancer	It was cross-sectional in design that the direction of the factors and the risk factors of anxiety disorders could not be established. The small sample size limited the power of the study to detect any other factors particularly involving the caregiving process such as duration of care that could have significance to the anxiety disorders
Khanjari et al. (2014) [35]	Iran	To explore how family caregivers of women with breast cancer in Iran describe the areas in life which are important to their quality of life (QoL) and to determine which areas in life that are influenced by having a family member with breast cancer	Descriptive and prospective cross-sectional study	88 family caregivers consisted of 29 (33%) men and 59 (67%) women with mean (SD) 41.1 (13.9).	A majority of family caregivers reported a high psychological impact described as experiences of shock and stress, feeling sadness and depressed, fear and anxiety, and having disturbed sleep. Furthermore, family caregivers stated other aspects which may have a negative impact on mental and emotional well-being but not covered, namely, descriptions of fear of recurrence, uncertainty of outcome, and worry about future and death. Many family caregivers reported a change in their relationship with their sick relative and that the disease tended to amplify existing problems. Religious aspects such as feeling a stronger connection to God as well as optimism and hope were expressed to be enhanced for some of the family caregivers	Family caregivers need support in dealing with the psychological strain related to the situation by acquiring information about cancer and its treatment and in how to communicate about their own concerns with their relative with cancer. Moreover, education and interventions from health care professionals would be beneficial in improving QoL for the families of patients	Sample size of the family caregivers is a limitation to the study. The use of semi-structured interviews may not be as adequate to capture a phenomenon as more in-depth interviews
Kusi et al. (2020) [4]	Ghana	To explore the caregiving motivations and experiences of family caregivers of patients living with advanced breast cancer	Exploratory descriptive phenomenological approach	15 caregivers. 7 were males while 8 were females with age range from 25 to 73 years.	Caregivers were involved in bathing, grooming, and cooking for patients. Their experiences further include symptom management such as the management of pain, lymphedema, wound, and evaluation of symptoms. Caregivers were the main providers of emotional support by offering patients with words of encouragement. They also experience financial burden by providing out-of-pocket money for treatment costs and other related non-medical costs	There is the need for home-based support programs to assist caregivers in their caring role especially in the area of symptom management and direct governmental social intervention programs (e.g., transportation to treatment facilities and drugs for patients) to resource-limited caregiving families of women with advanced breast cancer. The National Health Insurance Scheme should be expanded to fully cover breast cancer treatment to women and their family caregivers	The findings cannot be generalized based on the sample selection (caregivers of only advanced breast cancer patients). The study was limited to a single site

## Results

### Literature search results

Only articles that had reported on family caregivers of breast cancer patients in low- and middle-income countries were included. The initial search returned 1781 records. A total of 430 duplicates were removed using the Mendeley Desktop (version 1.19.4). The remaining 1351 records were screened for titles and abstracts by the two independent reviewers (GK and KBM). We then excluded 1225 articles, and a full-text screening was done on the remaining 126 articles. Following the full-text screening, 107 were excluded because they did not meet the inclusion criteria as detailed in the PRISMA flow diagram. At the end of the screening procedure, 19 peer-reviewed citations remained for final inclusion in the review [3, 4, 6, 10, 15, 17–19, 23–33].

**Fig. 1:**
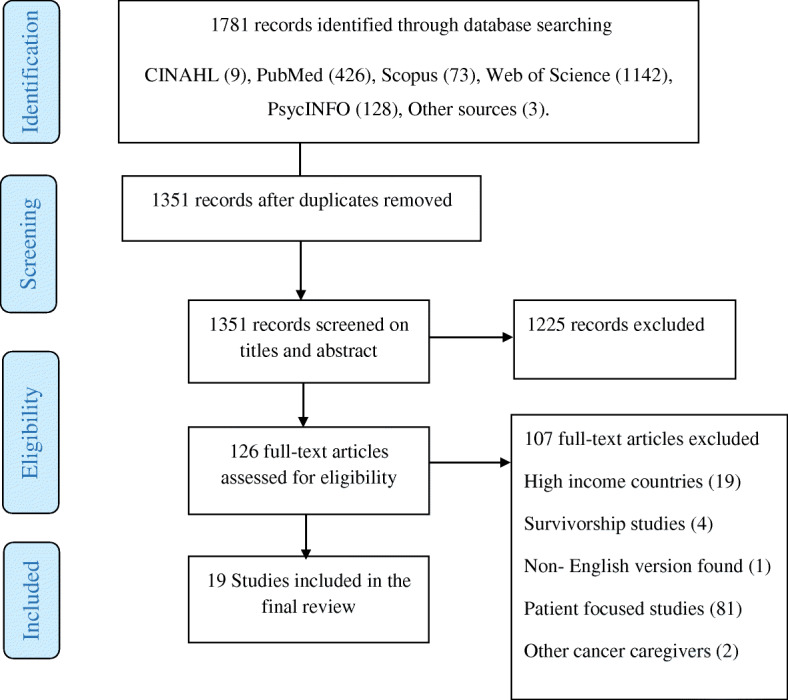
PRISMA flow diagram showing the systematic screening process

A schema illustrating the screening process is shown in Fig. 1.

### Methodological characteristics of included studies

The majority of the included studies (*n* = 15) used quantitative approaches [3, 10, 15, 18, 19, 23–28, 30–33] and the remaining (*n* = 4) were qualitative studies [4, 6, 17, 29]. Studies were conducted in the following countries: Nigeria (*n* = 2), Malaysia (*n* = 3), Ghana (*n* = 1), China (*n* = 3), Iran (*n* = 6), Turkey (*n* = 1), Mexico (*n* = 1), Indonesia (*n* = 1), and India (*n* = 1).

A total of 2330 family caregivers were included in the studies. The sample sizes within the included studies ranged from 7 caregivers [17, 29] to 384 caregivers [26]. The disease severity of the patients being cared for by the caregivers was only stated in 10 studies [4, 15, 24, 26, 28–32, 34].

Table 1 shows the summary of the included studies on caregivers’ experiences of women diagnosed with breast cancer.

### Quality appraisal or assessment of included studies

Quality appraisal of the included works of literature using MMAT ranged from moderate (50%) to strong quality (100%). Two studies were rated as strong quality [4, 6]. Fifteen studies were scored as moderate-strong quality [15, 18, 19, 23–33, 35], and the remaining two studies [3, 17] were scored as moderate. None of the included studies was excluded based on their quality assessment score. The level of agreement of quality ratings between the two independent raters ranged from strong [kappa 0.79; 95% (0.4–1.2); *p* = 0.001] to excellent agreement [kappa 1.0; 95% (0.9–1.0); *p* =0.001]. Table 2 shows the quality assessment of the included studies.

**Table 2:** Quality appraisal of included papers and their Mixed Methods Appraisal Tool (MMAT) score

		Qualitative				Quantitative					
Author, year	Sources relevant to address research question	Analysis Process relevant to address research question	Findings adequately derived from data	Interpretation of results sufficiently substantiated by data	Sampling strategy reduce selection bias	Measuring appropriate for intervention /outcome	Sample representativeness	Acceptable response rate	Total points	Score	Quality
Vahidi et al. (2016) [3]					0	0	1	1	(2/4)	50	Moderate
Gabriel, Aluko, and Okeme (2019) [15]					0	1	1	1	(3/4)	75	Moderate-strong
Zhu et al. (2014) [31]					0	1	1	1	(3/4)	75	Moderate-strong
Sahadevan et al. (2019) [26]					0	1	1	1	(3/4)	75	Moderate-strong
Jaafar et al. (2014) [24]					0	1	1	1	(3/4)	75	Moderate-strong
Gabriel and Mayers. (2019) [27]					0	1	1	1	(3/4)	75	Moderate -strong
Giray and Akyuz (2019) [28]					0	1	1	1	(3/4)	75	Moderate-strong
Moreno-Gonzalez et al. (2019) [29]	0	1	1	1					(3/4)	75	Moderate-strong
Hashemi-Ghasemabadi et al. (2016) [6]	1	1	1	1					(4/4)	100	Strong
Mahadevan et al. (2013) [32]					0	1	1	1	(3/4)	75	Moderate-strong
Bahrami and Farzi (2014) [18]					1	1	0	1	(3/4)	75	Moderate-strong
Wulandari et al. (2017)	0	1	0	1					(2/4)	50	Moderate
Yeung et al. (2018) [30]					0	1	1	1	(3/4)	75	Moderate-strong
Heidari Gorji et al. (2012)					0	1	1	1	(3/4)	75	Moderate-strong
Yuanyuan An et al. (2019)					0	1	1	1	(3/4)	75	Moderate-strong
Nejad et al. (2016)					1	1	0	1	(3/4)	75	Moderate-strong
Din et al. (2017) [25]					0	1	1	1	(3/4)	75	Moderate-strong
Khanjari et al. (2014) [35]					0	1	1	1	(3/4)	75	Moderate-strong
Kusi et al. (2020) [4]	1	1	1	1					(4/4)	100	Strong
	*k*; 0.9 (0.6–1.2) *p* < 0.05	*k*; 0.8 (0.5–1.1) *p* < 0.001	*k*; 1.0 (0.9–1.0) *p* = 0.001	*k*; 0.9 (0.7–1.1) *p* < 0.05	*k*; 0.79 (0.4–1.2) *p* < 0.001	*k*; 1.0 (0.9–1.0) *p* < 0.001	*k*; 1.0 (0.9–1.0) *p* < 0.001	*k*; 0.8 (0.4–1.2) *p* = 0.001			

Indicators: 0 criteria not met, 1 criteria met. Scale 1, 25% (one criterion met); scale 2, 50% (two criteria met); scale 3, 75% (three criteria met); scale 4, 100% (all criteria met)

k; Cohen’s kappa coefficient, 95% CI; 95% Confidence Interval, *p*; *p*-value

### Findings on the caregivers’ experiences

The synthesis of the included articles showed that caregivers’ experiences fell into 10 main categories, namely, (1) caregivers’ motivation, (2) roles of family caregivers, (3) quality of life among caregivers, (4) physical burden of caregiving, (5) psychological burden of caregiving, (6) disruption in social life, (7) economic burden of caregiving, (8) need for support, (9) interventions for improving caregiver experience, and lastly (10) coping. These findings are described in the ensuing paragraphs.

#### Caregivers’ motivation

Three studies focused on the caregivers’ motivation for delivering care to women with breast cancer [4, 6, 30]. In the first study, caregivers cited a sense of responsibility and commitment as forms of motivation for their caregiving roles [6]. One Ghanaian study reported that family and sociocultural obligations prompted family members, especially females, to assume the caregiving role for women with breast cancer [4]. However, two studies conducted in Ghana [4] and China [30] reported that family-oriented cultural norms also prompted males such as spouse caregivers to assume the role of primary caregivers.

#### Roles of family caregivers

Four studies emphasized the role of caregivers in the management of breast cancer [3, 4, 26, 29]. Vahidi et al. [3] and Kusi et al. [4] suggested that caregivers played key roles in assisting patients with activities of daily living and the administration of medications. The studies also showed that caregivers played roles in assisting with treatment decision-making and symptom management [3, 4, 26, 29]. Moreno-González et al. [29] and Kusi et al. [4] also emphasized that caregivers managed specific symptoms such as breast wound and lymphedema, evaluation of symptoms, and management of pain. One study also reported that caregivers were the primary source of psychosocial, spiritual, and financial support for women with breast cancer [4].

#### Quality of life among caregivers

Seven articles gave an account of the quality of life of caregivers. The reports indicated that caregivers of women diagnosed with breast cancer had low levels of quality of life as compared to the general healthy population [3, 15, 23, 26, 28, 31, 33]. One study reported that the patient correlates of poor quality of life among family caregivers were advanced-stage breast cancer, poor functional status, frequent hospitalization, longer duration of breast cancer [15], lack of transportation [3], and high symptom burdens such as wound [31] and postmastectomy lymphedema [28]. Further, An et al. [23] also cited that higher levels of anxiety and depression were associated with poor quality of life among caregivers. Lastly, three studies highlighted that lack of formal support services that characterized developing countries also resulted in poor quality of life among caregivers [3, 10, 33].

#### Physical burden of caregiving

Four studies emphasized that caregivers reported a moderate to severe decline in physical health [6, 10, 17, 31]. Altered sleep patterns and hypertension were the frequently reported physical symptoms experienced by the caregivers [6, 10, 17]. Zhu et al. also highlighted that low family income and increasing age are predictors of physical burden among caregivers [31].

#### Psychological burden of caregiving

The majority of the included studies reported that psychological burden was the most frequent stressor that caregivers encountered [6, 10, 17, 25–27, 29–33]. According to Khanjari et al. [10], more than 70% of family caregivers experienced severe psychological impacts 6 months following breast cancer diagnosis and the assumption of the caregiving role. These studies also identified some conditions described as psychological burdens among the caregivers. Six studies cited depression as a common psychological burden among caregivers [10, 24, 30–33]. Studies cited that factors such as age [32], male gender, altered sleeping pattern [10, 17, 30, 31], longer duration of caregiving [24, 26, 32], lower educational level, not sharing caregiving responsibilities [32], and decreased functional status of patients [24, 32] were significantly associated with stressors such as depression among caregivers. Additionally, anxiety had also been identified as a common psychological distress that is experienced by caregivers [17, 25]. According to Din et al., about one third of caregivers suffered from anxiety-related disorders [25]. Furthermore, Din et al. [25] reported that longer caregiving duration and absence of shared caregiving were significantly associated with anxiety disorders. Moreover, four of the included studies reported that anxiety among caregivers was caused by the uncertainties of breast cancer outcomes, fear of recurrence, and poor financial supports available to caregivers [10, 17, 25, 26, 29]. Several emotional traumas such as fear, shock, anger, and sadness were also cited by caregivers in three studies [6, 26, 29].

#### Disruption in social life

Hashemi-Ghasemabadi et al. [6] reported that caregivers experienced a loss of normal life. This study also reported that caregivers experienced deteriorating relationships with other family members. Some caregivers cited that they felt isolated and lacked support from family and friends, which increased the burden associated with their caregiving roles [6].

#### Economic burden of caregiving

Eight papers examined the economic burden associated with caregiving around breast cancer [3, 6, 10, 15, 17, 24, 27, 29]. Two studies reported that caregivers usually decreased their working hours or lost paid jobs as a result of the caregiving role [17, 24]. It was also reported that even in conditions where caregivers still engaged in paid jobs, high treatment costs coupled with the absence of fully financed healthcare system that exists in LMICs created a high economic burden for family caregivers [15, 17, 24, 27, 29]. The synthesized findings also showed that lack of basic financial resources [15, 29] and inadequate income for meeting caregiving demands [3, 6, 10, 27] also resulted in financial burden for caregivers. Further, Gabriel and Mayers [27] suggested that given the extreme financial burden that is faced by caregivers in developing countries, educational interventions might be ineffective in improving the financial well-being among caregivers.

#### The need for support

The need for social support was the most frequent need that was cited by the caregivers across six included studies [6, 17, 25, 29–31]. Caregivers cited that they had not received adequate social support from their families and friends [17, 25, 31]. Specifically, caregivers described the need for sharing caregiving responsibilities with other family members and friends [6, 25, 30, 31].

Nonetheless, Yeung et al. [30] and Vahidi et al. [3] reported that seeking support from families and friends among spouse caregivers may result in increased emotional distress as it may be culturally interpreted as a sign of weakness and lack of self-confidence. Further, in three studies, caregivers also stressed the importance of support from the formal care systems to help them manage patients’ symptoms in the home setting [6, 17, 29].

#### Interventions for improving the caregiver’s experience

Three articles examined interventions to support family caregivers [18, 19, 27]. Largely, all the studies reported significant improvement in the quality of life after psycho-educational intervention. Particularly, the improvements in caregivers’ quality of life were related to measures such as the emotional aspect of caregiving. In the first study [27], it was reported that caregivers were given psycho-educational interventions to improve their quality of life. Caregivers in the interventional group received 6 weeks of information about adjustment to the role of family caregivers and strategies to deal with the emotional aspects of caregiving. This quasi-experimental study reported that caregivers in the interventional group reported better quality of life after the 6 weeks [27]. It was reported that the provision of informational support aided in decreasing caregiving burden among the caregivers. However, the intervention did not affect the financial well-being of caregivers.

The second education intervention study [18] examined the effect of a supportive educational program on the caring burden and quality of life of family caregivers. The intervention group in this study reported a clear increase in caregivers’ knowledge about breast cancer management, physical, psychological, spiritual, and environmental dimensions of health. Lastly, the third study [19] evaluated the effect of an educational and telephone follow-up on caregiver burden. Results from this showed that caregiver burden decreased significantly among the intervention group.

#### Coping

Three of the included studies reported on coping strategies used by family caregivers [10, 17, 29]. These studies reported that religious coping such as putting one’s faith in God was vital in improving the quality of life among caregivers [10, 17, 29]. Two of the studies further reported that caregivers reported that being religious provided them with meaning in their caregiving roles [10, 17]. Further, one study also showed that previous knowledge on breast cancer aided caregivers to cope effectively in their caring role [29].

## Discussion

In this systematic review, key evidence on family caregivers of women diagnosed with breast cancer in LMICs has been highlighted. Reviews focusing on caregivers of women with breast cancer in LMICs are missing in the literature. The current systematic review, therefore, addressed this gap by adding to the knowledge in this area.

The current review demonstrated that family members, including male spouses, assumed the caregiving role for women diagnosed with breast cancer. Based on this finding, future research using comparative study designs should be conducted to examine how breast cancer caregiving differs among male and female caregivers. This systematic review further highlighted the roles that are played by family caregivers in providing physical, spiritual, emotional, and financial support to women with breast cancer in LMICs [3, 4]. Furthermore, studies in this review highlighted the significant role played by caregivers in symptom management [3, 4, 26]. Despite this important finding, only one of the included studies [4] provided information on how symptoms such as pain, lymphedema, and breast wound are managed in the home by the caregivers. The level of empirical evidence addressing how family caregivers manage symptoms in LMICs where there is evidence of limited formal support structures needs further exploration using qualitative methods.

The review identified a range of burdens that are encountered by caregivers. Caregivers reported challenges such as fear, depression, and hypertension [6, 25, 31]. Financial challenges such as lack of transportation, loss of a paid job, and high treatment cost were also fundamental sources of stress for caregivers across several studies in this review [3, 26, 27, 29]. This finding is expected, considering the financial burden that such caregiver roles put on families. This is because these caregivers are already overburdened by the lack of formal support services and poor economic status in LMICs. Therefore, these identified challenges encountered by the caregivers in LMICs in their caregiving roles represent areas in the caregivers’ lives that need to be addressed in policy formulation.

Also, it was reported that disease severity and declining functional status impacted the quality of life of the caregivers [3, 15, 26, 31]. As such, how the caregivers’ challenges change according to the progression of breast cancer is an important topic for further exploration in future longitudinal studies.

The findings of this review also showed that educational and psychological interventions could prove as relevant tools in improving the wellbeing of family caregivers [18, 19, 27]. Accordingly, it would be important for future study to focus on the development of educational and supportive interventions for family caregivers to help address their challenges.

One of the most striking gaps observed in this systematic review is the lack of qualitative studies on the caregivers of women with breast cancer in LMICs. Only four studies explored the experiences and challenges that were faced by the caregivers in their caregiving roles using qualitative designs [4, 6, 17, 29]. This finding therefore offers opportunity for further qualitative works into breast cancer caregiving in LMICs. This method of enquiry will produce a rich, detailed, and rigorous data on the phenomenon by allowing participants to naturally share their lived experiences in their setting.

Lastly, there are a few studies that explored other aspects of caregiver wellbeing such as disruption in social life, coping measures, and intervention for caregivers [6]. Therefore, a need exists to focus future studies on these areas of the caregiver wellbeing.

## Strengths and limitations

Some limitations of this systematic review are worth noting. First, this review found a relatively small number of relevant studies. With a publication period from January 2000 to March 2020, only 19 studies that had reported on family caregivers of breast cancer patients were identified and synthesized. This may hinder the generalizability of the research findings. However, with expanded attention to breast cancer caregivers in LMICs, this review can drive future research and also inform policy. Further limitation is language restriction. Non-English language articles were excluded due to the limited capacity to access professional language services. This is challenging since family caregiving in breast cancer is socio-cultural and context-specific. Excluding articles in non-English languages may have resulted in a limited understanding of the phenomenon under review and therefore poses a risk of bias in extrapolating the results to a broader population. Further, not considering studies that had been published in the developed countries might have resulted in the exclusion of relevant studies.

In addition, it was not feasible to synthesize the results in a meta-analysis as the included studies were clearly heterogenous. For instance, the included studies used different methods such as correlational studies, cross-sectional designs, and self-reported data to obtain caregivers’ reported experiences. It was however decided a priori to include all studies regardless of their study design as the aim of this study was to investigate the diversity of studies reporting on caregivers of women living with breast cancer in LMICs, thus obtaining a broad perspective of the phenomenon under review.

The strength of this systematic review includes the use of an MMAT tool for the quality appraisal by two independent reviewers. Also, we are the first to systematically map evidence and report on the experiences of family caregivers of breast cancer patients in low- and middle-income countries.

## Conclusions

The incidence of breast cancer is increasing in LMICs. This has serious implications for family caregivers. The burden associated with the caregiving role is further amplified by limited availability and access to palliative services and formal structures to support caregivers in their caring roles. Also, due to factors such as financial constraints, there may be a lack of social protection policies for breast cancer caregivers. The high psychological and financial burdens associated with caregiving in developing countries create the need to raise awareness about the neglected needs of family caregivers.

Enhanced support for caregivers should be highlighted as a priority in LMICs. There are numerous gaps in policy and education about caregivers that need to be addressed. The available evidence in this review does not comprehensively address how caregiver challenges can be reduced. Therefore, further research is needed to generate empirical data to inform an evidence-based approach to addressing family caregivers’ challenges in LMICs.

## Contribution of the paper

### What is known about this topic?

- Breast cancer is a common malignancy among women in LMICs.
- There is a transition of breast cancer treatment from the in-patient setting to the out-patient setting.

### What this paper adds:

- Evidence on family caregivers of breast cancer patients in LMICs.
- Identify the limited evidence and the existing gaps in research related to breast cancer caregiving that urgently needs to be addressed.
- Family caregivers play a key role in providing home care for breast cancer patients in LMICs.
- Family caregivers experience challenges in their caregiving role.

## Supplementary information

**Additional file 1: Table S1.** Search strategy.

## Data Availability

All data generated or analyzed during this study are included in this published article (and its supplementary information files). The data can be accessed at figshare data repository through 10.6084/m9.figshare.12089205.

## Abbreviations

LMICs: Low-and middle-income countries
MMAT: Mixed Methods Appraisal Tool
