# Supplementary material for: The experiences of family caregivers living with breast cancer patients in low-and middle-income countries: a systematic review
Source: Syst Rev. 2020 Jul 23;9:165. doi: 10.1186/s13643-020-01408-4 (PMC7379811; doi:10.1186/s13643-020-01408-4)
Supplement: Supplementary file 1 — Additional file 1: Table S1. Search strategy. [file 13643_2020_1408_MOESM1_ESM.docx]

|  | SEARCH TERMS | DATE | PUBMED | CINAHL | PSYCINFO | SCOPUS | WEB OF SC. | Other Source (Google Scholar) |
| --- | --- | --- | --- | --- | --- | --- | --- | --- |
| S1 | experienc*[tw] OR practic*[tw] OR involvement* [tw] OR participat* [tw] OR understanding [tw] | March, 2020 | 2350066 | 11,773 | 559,560 | 3,779,032 | 3,774,66 |  |
| S2 | quality of life[tw] OR Quality of life [tw] OR life quality[tw] OR quality life[tw] OR quality-of-life[tw] | March, 2020 | 314435 | 4,517 | 112,489 | 371,825 | 354,390 |  |
| S3 | home?Care[tw] OR home-care[tw] OR home aid[tw] OR home based care [tw] | March, 2020 | 421613 | 1,389 | 19814 | 28,996 | 81375 |  |
| S4 | physical burden*[tw] OR pcychological burden*[tw] OR economic burden*[tw] OR social burden*[tw] OR financial burden*[tw] | March, 2020 | 203098 | 333 | 2,253 | 14120 | 62546 |  |
| S5 | cop*[tw] OR coping Skill*[tw]OR coping behavior*[tw] OR coping[tw] | March, 2020 | 39382 | 2755 | 27,790 | [54,219](https://www-scopus-com.ezp.twu.edu/search/history/results.uri?origin=searchhistory&shid=12) | 52,892 |  |
| S6 | stress*[tw] OR Job-related stress*[tw] OR Job stress*[tw] OR work-related stress*[tw] OR work stress*[tw] | March, 2020 | 739821 | 4,534 | 114,757 | [1,245,809](https://www-scopus-com.ezp.twu.edu/search/history/results.uri?origin=searchhistory&shid=8) | 1,282,225 |  |
| S7 | caregiv*[mh] OR carer*[mh] OR care giver*[mh]OR spouse caregiver*[mh] OR family caregiv* [mh] | March, 2020 | 69062 | 2,177 | 28,417 | 3,408 | 60286 |  |
| S8 | breast neoplasm*[mh] OR breast cancer*[mh] OR malignant breast cancer*[mh] OR cancer of breast [mh]OR cancer of the breast [mh] | March, 2020 | 274915 | 592 | 7,782 | [248,765](https://www-scopus-com.ezp.twu.edu/search/history/results.uri?origin=searchhistory&shid=14) | 325,031 |  |
| S9 | low-income countr*[tw] OR lower-middle-income countr*[tw] OR upper- middle- income countr*[tw] OR emerging countr* [tw]OR emerging nation* [tw]OR emerging population* [tw]OR developing countr*[tw]OR developing nation*[tw]OR developing population*[tw] OR developing world [tw]OR less developed countr*[tw]OR less developed nation*[tw] OR less developed population* [tw]OR transitional countr*[tw] | March, 2020 | 35954 | 25626 | 983523 | 4,956,427 | 11,834,208 |  |
| S10 | S1 OR S2 OR S3 OR S4 OR S5 OR S6 | March, 2020 | 3524813 | 7,858 | 664512 | 4756769 | 5104644 |  |
| S11 | S7 AND S9 AND S10 | March, 2020 | 51173 | 404 | 21301 | 2639 | 45,053 |  |
| S12 | S8 AND S11 | April, 2020 | 425 | 9 | 128 | 73 | 1142 | **3** |
